# Supplementary material for: Higher-order Granger reservoir computing: simultaneously achieving scalable complex structures inference and accurate dynamics prediction
Source: Nat Commun. 2024 Mar 20;15:2506. doi: 10.1038/s41467-024-46852-1 (PMC10954644; doi:10.1038/s41467-024-46852-1)
Supplement: Supplementary file 1 — Supplementary Information [file 41467_2024_46852_MOESM1_ESM.pdf]

Contents

Supplementary Note 1 Additional Information on the HoGRC Framework 2
1.1 Proof of Proposition 1 2
1.2 Search algorithms for higher-order neighbors 3
1.3 An intuitive example for higher-order RC 5
1.4 Direct and indirect causality 6
Supplementary Note 2 Effects in Other Common Differential Dynamical Systems 8
2.1 Effects in several common chaotic systems 8
2.2 The hyperchaotic system with four wings 10
Supplementary Note 3 Hyperparameter settings for different models 12
Supplementary Note 4 Some Supplementary Experiments 15
4.1 Lorenz63 system 15
4.2 Lyapunov exponents of the coupled Lorenz63 system 15
4.3 Higher-Order Neighbor Inference in Network Dynamical Systems 17
4.4 Structure information of the UK power grid 17
4.5 System structure inference with noise data 17
4.6 Influence of coupling strength 18
4.7 Higher-order Kuramoto model with phase lags 18
Supplementary Note 5 NDCN and TPI Methods for Network Dynamical Systems 20

Supplementary Note 1. Additional Information on the HoGRC Framework

1.1. Proof of Proposition 1

We know that the feature information stored in the hidden state $\mathbf{r}$ is influenced by the input matrix $\mathbf{W}_{\text{in}}$ and the adjacency matrix $\mathbf{A}$. Given that the final model is represented by a linear transformation $\mathbf{W}_{\text{out}}\mathbf{r}$, it becomes apparent that the higher the dimension of the linear space expanded by hidden state $\mathbf{r}$, the more complex nonlinear features of model input $\mathbf{x}$ can be captured. Based on this, we give the proof of proposition 1 as follows.

Proof. For any $h_{HoGRC} \in \mathscr{H}$, if its input matrix is $\tilde{\mathbf{W}}_{\text{in}}$, adjacency matrix is $\tilde{\mathbf{A}}$, and output matrix is $\tilde{\mathbf{W}}_{\text{out}}$, and it may be considered that $l = 1$, then the hidden dynamics of h_{HoGRC} is

$$\tilde{\mathbf{r}}(t) = \tanh(\tilde{\mathbf{A}}\tilde{\mathbf{r}}(t-1) + \tilde{\mathbf{W}}_{\text{in}}\tilde{\mathbf{W}}_{\text{out}}\tilde{\mathbf{r}}(t-1) + \sigma_{\text{b}})$$

where $\tilde{\mathbf{W}}_{\text{out}}\tilde{\mathbf{r}}(t) = \mathbf{x}(t)$, and a vector addition with σ_b indicates the addition of σ_b to each dimension of this vector. We take $\mathbf{A} = \tilde{\mathbf{A}}$, and take $\mathbf{W}_{\text{in}}$ and $\mathbf{W}_{\text{out}}$ to satisfy $\mathbf{W}_{\text{in}}\mathbf{W}_{\text{out}} = \tilde{\mathbf{W}}_{\text{in}}\tilde{\mathbf{W}}_{\text{out}}$, then $h_{\text{HoGRC}} \in \mathscr{H}_{\text{RC}}$, so we have

$$\mathscr{H}_{\text{HoGRC}} \subseteq \mathscr{H}_{\text{RC}}.$$

Since the research in reference [1] shows that the upper bound of generalization error is positively related to the upper bound of hidden state $\mathbf{r}$. So we only need to prove that the upper bound of $\tilde{\mathbf{r}}$ in HoGRC is less than the upper bound of $\mathbf{r}$ in RC. Because the hidden state satisfies

$$\begin{aligned}
\|\mathbf{r}(t)\|_2 &= \|\tanh(\mathbf{A}\mathbf{r}(t-1) + \mathbf{W}_{\text{in}}\mathbf{x}(t) + \sigma_{\text{b}})\|_2 \\
&\leq \sqrt{n}\|\tanh(\mathbf{A}\mathbf{r}(t-1) + \mathbf{W}_{\text{in}}\mathbf{x}(t) + \sigma_{\text{b}})\|_\infty \\
&\leq \sqrt{n}\tanh(\|\mathbf{A}\mathbf{r}(t-1)\|_\infty + NB\|\mathbf{W}_{\text{in}}\|_\infty + |\sigma_{\text{b}}|),
\end{aligned}$$

where n is the number of nodes in reservoir network, N is the dimension of $\mathbf{x}$, and B is the upper bound of the infinite norm of $\mathbf{x}$. And

$$\begin{aligned}
\|\mathbf{A}\mathbf{r}(t-1)\|_\infty &= \|\mathbf{A}\tanh(\mathbf{A}\mathbf{r}(t-2) + \mathbf{W}_{\text{in}}\mathbf{x}(t-1) + \sigma_{\text{b}})\|_\infty \\
&\leq \|\mathbf{A}(\mathbf{A}\mathbf{r}(t-2) + \mathbf{W}_{\text{in}}\mathbf{x}(t-1) + \sigma_{\text{b}})\|_\infty \\
&\leq \|\mathbf{A}^2\mathbf{r}(t-2)\|_\infty + \|\mathbf{A}\|_\infty(NB\|\mathbf{W}_{\text{in}}\|_\infty + |\sigma_{\text{b}}|),
\end{aligned}$$

so we can get

$$\begin{aligned}
\|\mathbf{r}(t)\|_2 &\leq \sqrt{n}\tanh(\|\mathbf{A}\mathbf{r}(t-1)\|_\infty + NB\|\mathbf{W}_{\text{in}}\|_\infty + |\sigma_{\text{b}}|) \\
&\leq \sqrt{n}\tanh\left(\|\mathbf{A}^2\boldsymbol{r}(t-2)\|_\infty + (NB\|\mathbf{W}_{\text{in}}\|_\infty + |\sigma_{\text{b}}|)(\|\mathbf{A}\|_\infty + \|\mathbf{A}^2\|_\infty\right) \\
&\leq \cdots \\
&\leq \sqrt{n}\tanh\left(\|\mathbf{A}^{(t)}\mathbf{r}(0)\|_\infty + (NB\|\mathbf{W}_{\text{in}}\|_\infty + |\sigma_{\text{b}}|) \times \frac{\|\mathbf{A}\|_\infty^{(t)} - 1}{\|\mathbf{A}\|_\infty - 1}\right).
\end{aligned}$$

We choose $\mathbf{0}$ as the initial value of the hidden state, and for any $t = 1, 2, \cdots T$, we have

$$\|\mathbf{r}(t)\|_2 \leq R_u = \sqrt{n} \tanh\left((NB\|\mathbf{W}_{\text{in}}\|_\infty + |\sigma_{\text{b}}|) \times \frac{\|\mathbf{A}\|_\infty^{(T)} - 1}{\|\mathbf{A}\|_\infty - 1} \right).$$

Since $\tilde{\mathbf{W}}_{\text{in}}$ is a sparse version of $\mathbf{W}_{\text{in}}$ and $\tilde{\mathbf{A}}$ is a sparse version of $\mathbf{A}$, then $\|\tilde{\mathbf{W}}_{\text{in}}\|_\infty \leq \|\mathbf{W}_{\text{in}}\|_\infty$ and $\|\tilde{\mathbf{A}}\|_\infty \leq \|\mathbf{A}\|_\infty$, so the upper bound of hidden state in HoGRC is smaller than that in RC. Therefore, the HoGRC method has a smaller upper bound of the generalization error, that is,

$$GE_{\text{u}}(h_{\text{HoGRC}}) \leq GE_{\text{u}}(h_{RC}),$$

where $h_{\text{HoGRC}} \in \mathscr{H}_{HoGRC}$ and $h_{RC} \in \mathscr{H}_{RC}$.

In fact, the results of the main text demonstrate that the proposed method offers significant advantages in the extrapolation prediction task. Additionally, experimental evidence confirms that our method can learn more real dynamic characteristics from the perspective of experiments, so it has less generalization error rather than just the upper bound.

1.2. Search algorithms for higher-order neighbors

The observational data of dynamical systems do not contain spatial information, so it is necessary to infer higher-order neighbor information in the first task. According to Definition 3 in the main text, we design an efficient greedy algorithm to search for the higher-order neighbors of any node. We search for true higher-order neighbors in order from high-dimensional to low-dimensional, and in the process ensure that there is no inclusion relationship between complexes in the candidate neighbor set (Definition 2 in the main text).

Before giving the search algorithm, we first give the following three definitions.

Definition 1. *The inclusion relationship of the candidate neighbor set. Consider two candidate neighbor sets $\mathscr{C}_i = \{\mathbf{c}_{1,i}, \cdots, \mathbf{c}_{K_i,i}\}$ and $\mathscr{C}_j = \{\mathbf{c}_{1,j}, \cdots, \mathbf{c}_{K_j,j}\}$. If for any complex $\mathbf{c}_i \in \mathscr{C}_i$, there exists a complex $\mathbf{c}_j \in \mathscr{C}_j$ such that $\mathbf{c}_i \subseteq \mathbf{c}_j$, then $\mathscr{C}_i \subseteq \mathscr{C}_j$. And if $\mathscr{C}_i \neq \mathscr{C}_j$, then $\mathscr{C}_i \subset \mathscr{C}_j$.*

Definition 2. *Complex dimensionality reduction. Consider a p-dimension complex $\mathbf{c} = \{u_1, \cdots, u_p\}$, then reducing the dimension of the p-dimensional complex gets p complexes of dimension $(p-1)$, i.e.,*

$$\{\{u_2, u_3, \cdots, u_p\}, \{u_1, u_3, \cdots, u_p\}, \cdots, \{u_1, \cdots, u_{i-1}, u_{i+1}, \cdots, u_p\}, \cdots, \{u_1, u_2, \cdots, u_{p-1}\}\}.$$

Definition 3. *Remove redundant complexes. Consider a candidate complex set $\mathscr{C}$, for any $\mathbf{c} \in \mathscr{C}$, if there exists $\tilde{\mathbf{c}} \in \mathscr{C}$ satisfy $\mathbf{c} \subset \tilde{\mathbf{c}}$, then remove $\mathbf{c}$ from $\mathscr{C}$.*

The operation of Definition 3 is to make the candidate complex set available as a higher-order neighbor set. On the basis of this definition, Algorithm 1 shows the pseudocode of this search strategy, where lines 4 to 14 represent the process of removing invalid complexes, and lines 15 to 23 represent the complex dimensionality reduction process. It is not difficult

Algorithm 1: Search algorithms for higher-order neighbors

Data: The candidate neighbor set of node u $\mathscr{C}_0 = \{\mathbf{c}_{1,0}, \cdots, \mathbf{c}_{K_0,0}\}$, time series data $x_1(t), x_2(t), \cdots, x_N(t)$.

Result: The higher-order neighbors of node u $\mathscr{S}_u = \{\mathbf{s}_{u,1}, \cdots, \mathbf{s}_{u,D_u}\}$.

$i \leftarrow 0$, $a \leftarrow 1$;
Set a suitable threshold ϵ_{e};
while $a > 0$ **do**
    while $a > 0$ **do**
        $a \leftarrow 0$;
        for $j \leftarrow 0$ **to** K_i **do**
            if $e_{\{\mathbf{c}_{1,i}, \cdots, \mathbf{c}_{K_i,i}\}}(u) + \epsilon_e \geq e_{\{\mathbf{c}_{1,i}, \cdots, \mathbf{c}_{j-1,i}, \mathbf{c}_{j+1,i}, \cdots, \mathbf{c}_{K_i,i}\}}(u)$ **then**
                $\mathscr{C}_{i+1} = \{\mathbf{c}_{1,i+1}, \cdots, \mathbf{c}_{K_{i+1},i+1}\} \leftarrow \{\mathbf{c}_{1,i}, \cdots, \mathbf{c}_{j-1,i}, \mathbf{c}_{j+1,i}, \cdots, \mathbf{c}_{K_i,i}\}$;
                $i \leftarrow i + 1$;
                $a \leftarrow 1$;
                break;
            end
        end
    end
    Rearrange the elements in $\mathscr{C}_i$ from high order to low order;
    for $j \leftarrow 0$ **to** K_i **do**
        Reduce the dimensionality of the complex $\mathbf{c}_{j,i}$ to get the complex set $\mathscr{C}_{j,i}$;
        if $e_{\{\mathbf{c}_{1,i}, \cdots, \mathbf{c}_{K_i,i}\}}(u) + \epsilon_e \geq e_{\mathscr{C}_{j,i} \cup \{\mathbf{c}_{1,i}, \cdots, \mathbf{c}_{j-1,i}, \mathbf{c}_{j+1,i}, \cdots, \mathbf{c}_{K_i,i}\}}(u)$ **then**
            $\mathscr{C}_{i+1} = \{\mathbf{c}_{1,i+1}, \cdots, \mathbf{c}_{K_{i+1},i+1}\} \leftarrow \mathscr{C}_{j,i} \cup \{\mathbf{c}_{1,i}, \cdots, \mathbf{c}_{j-1,i}, \mathbf{c}_{j+1,i}, \cdots, \mathbf{c}_{K_i,i}\}$;
            $i \leftarrow i + 1$;
            $a \leftarrow 1$;
            break;
        end
    end
    Remove redundant complexes in $\mathscr{C}_i$;
end
$\mathscr{S} \leftarrow \mathscr{C}_i$;

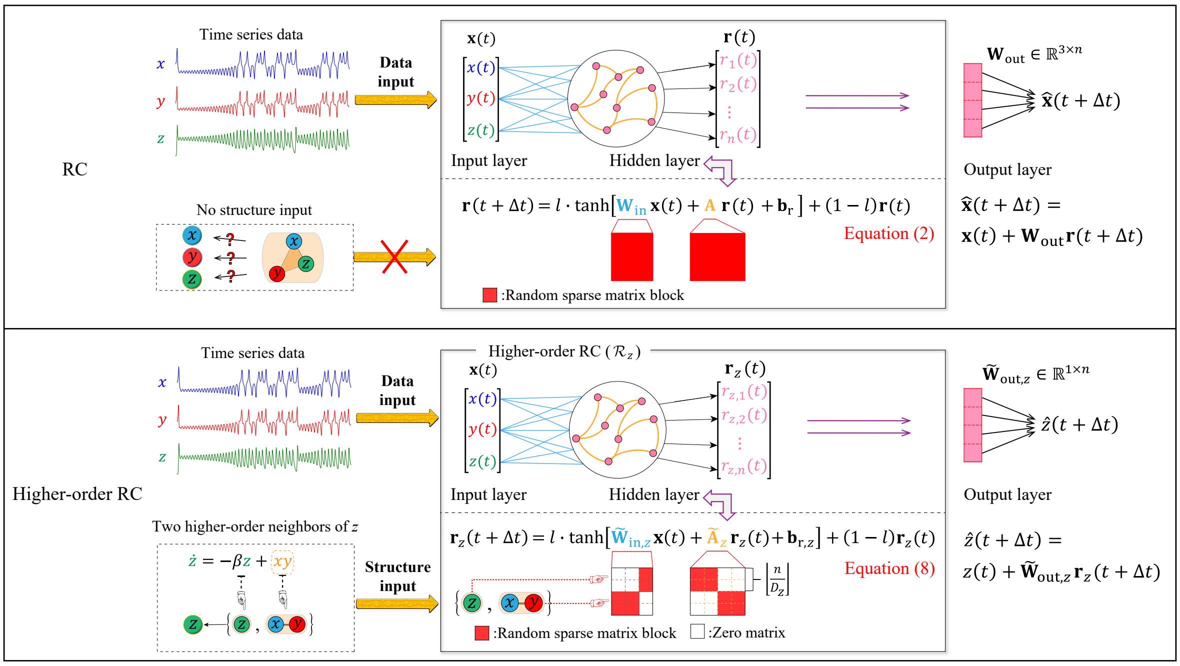

Supplementary Fig. 1: The difference between the RC with hidden dynamics of Equation (2) in the main text and the HoGRC with hidden dynamics of Equation (5) in the main text.

to find that $\mathscr{C}_i \supset \mathscr{C}_{i+1}$ during the execution of the algorithm, and finally we can find the true higher-order neighbor set $\mathscr{S}$.

Choosing an appropriate parameter ϵ_{e} is the key to accurately infer the higher-order neighbors of the system. Usually, ϵ_{e} is a fixed value for a specific node. In the experiment, we found that the experimental error of including the real higher-order neighbors is small, and the experimental error of not including the higher-order neighbors is large, so the dotted line in the figure in the main text can divide the error into two parts. In addition, the more accurate the modeled dynamics of HoGRC, the larger the selection range of the parameter ϵ_{e}, and the easier it is to infer the true higher-order neighbors. Of course, when the threshold selection is inappropriate, the inferred higher-order neighbors $\hat{\mathscr{S}}$ may be biased. But in most cases, $\hat{\mathscr{S}} \supset \mathscr{S}$, and this incomplete inference can still significantly improve the prediction ability of RC.

1.3. An intuitive example for higher-order RC

Here, we consider the Lorenz63 system as a simple example to illustrate the difference between higher-order RC and the traditional RC, and we also visually display this difference in Supplementary Fig. 1. The primary distinction between our method and traditional RC lies in the exploitation of the higher-order structural information to achieve the node-level prediction, with the details as follows.

- **RC**. In the traditional RC framework, both the input matrix $\mathbf{W}_{\mathrm{in}}$ and the adjacency matrix $\mathbf{A}$ are randomly initialized without incorporating any structural information as

visually shown in Supplementary Fig. 1. Actually, only a *single* RC with the readout layer is used for the prediction of the whole system state $\mathbf{x} = (x, y, z)$.

- **Higher-order RC**. We use the Lorenz63 system for illustration. Actually, we have a total of *three* sub-RC networks with higher-order structures because there are *three* state variables, viz. $x, y, z \in V$ for this system. Different sub-RC has different input and adjacency matrices, notably incorporating high-order structures. Particularly, we consider a specific node (state variable) $u = z$ in the Lorenz63 system, as shown in Supplementary Fig. 1. We write out

$$\dot{z} = f_3(x, y, z) = -\beta z + xy = g(x, y, z) = g_1(z) + g_2(x, y),$$

where $g_1(z) \triangleq -\beta z$, $g_2(x, y) \triangleq xy$, and $D_z \triangleq 2$. Consequently, according to Definitions 1 & 2 in the main text, the set of the higher-order neighbors of node z is $\mathscr{S}_z = \{\mathbf{s}_{z,1}, \mathbf{s}_{z,2}\} = \{\{z\}, \{x, y\}\}$.

As mentioned above, the set of the higher-order neighbors of node z is $\{\{z\}, \{x, y\}\}$ with $D_z = 2$. Thus, we obtain $\tilde{\mathbf{W}}_{\text{in},z} = [\psi^\top(z), \psi^\top[(x, y)]]^\top$ according to the notations set in Equation (6) of the main text, where the third column of $\psi^\top(z)$ and the first and the second columns of $\psi^\top[(x, y)]$ are the random sparse submatrices, and the remaining parts are zero submatrices. Moreover, we obtain $\tilde{\mathbf{A}}_z = \text{diag}\{\varphi(z), \varphi[(x, y)]\}$, which is a block diagonal matrix comprising two random sparse submatrices. Therefore, we encode this higher-order structural information into the input matrix $\tilde{\mathbf{W}}_{\text{in},z}$ and the adjacency matrix $\tilde{\mathbf{A}}_z$, yielding the Equation (5).

Finally, the following module is the readout layer through the matrix $\tilde{\mathbf{W}}_{\text{out},z}$, employed for the prediction of node z. And due to our approach operates at the node level, we can execute predictions for all nodes $\mathbf{x} = (x, y, z)$ in parallel.

Based on the known higher-order structures $\{\{z\}, \{x, y\}\}$ of node z, we can use higher-order RC achieve more precise dynamics prediction.

1.4. Direct and indirect causality

In our HoGRC framework, RC does not require exact learning of the system structure through GC, since our framework only focuses on coupling specific structures to maximize predictive performance. Therefore, in the inference task, both direct causality and indirect causality may be inferred. Despite this, our framework consistently and accurately infers the high-order structures in multiple experiments conducted in this study. We attribute the accuracy of our framework in the inferring tasks to several primary factors.

Firstly, many of the systems analyzed in our work display complex chaotic features, spurious causality can thereby lead to the rapid accumulation of errors, significantly reducing the predictive capacity of our framework in comparison to true causality.

Furthermore, the systems we study typically contain self-loops, as depicted by the node Y in Supplementary Fig. 2a. It allows us to accurately capture the direct neighbors rather than the indirect ones. In Supplementary Fig. 2a, the set of direct neighbors of node Z

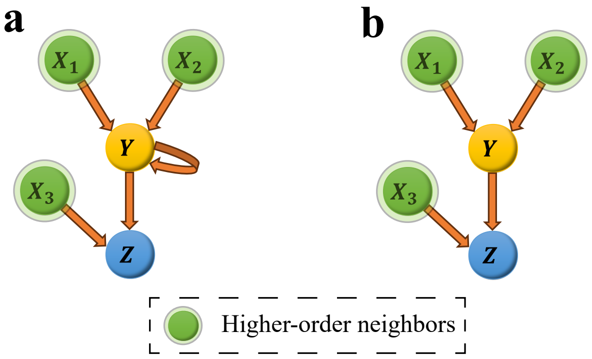

Supplementary Fig. 2: Detection of causal links from X and Y to Z. a The node Y has a self-loop edge. **b** The node Y does not have a self-loop edge.

is $O_Z = \{X_3, Y\}$, while the set of direct neighbors of Y is $O_Y = \{X_1, X_2, Y\}$, where X_1, X_2, X_3 are higher-order nodes defined using the simplicial complex in our work, and Y and Z are the first-order nodes. Considering the indirect neighbors $\{X_3\} \cup O_Y$, it holds that $O_Z = \{X_3, Y\} \subseteq \{X_3\} \cup O_Y$. Since $Y \in O_Y$, we cannot exclude Y from the inference process. Otherwise, this may result in a poor prediction of Y solely based on $\{X_1, X_2\}$, as well as a poor prediction of Z based on $\{X_1, X_2, X_3\}$. Therefore, we ultimately eliminate neighbors X_1 and X_2, which do not directly contribute to the prediction of Z, to obtain the true higher-order structure $O_Z = \{X_3, Y\}$.

When Y has no self-loop, the situation is different. As shown in Supplementary Fig. 2b, since $O_Y = \{X_1, X_2\}$, both $C_1 = \{X_1, X_2, X_3\}$ and $C_2 = \{X_3, Y\}$ can theoretically predict Z well. Therefore, the current HoGRC framework cannot distinguish this case. To address this issue, we extend our HoGRC framework to further identify the indirect causality via combining with the existing methods. In particular, we propose two strategies: (1) conditional Granger causality and (2) further causal identification. To verify the effectiveness of these two strategies, we constructed a dynamical system with a local structure given by Supplementary Fig. 2b, which reads

$$
\begin{aligned}
\dot{x}_1 &= 10(x_2 - x_1), \\
\dot{x}_2 &= 28x_1 - x_2 - x_1x_3, \\
\dot{x}_3 &= x_1x_2 - 8/3x_3, \\
\dot{y} &= \alpha_y - \beta_1 x_1 + \beta_2 x_2, \\
\dot{z} &= \alpha_z - \gamma_1 y + \gamma_2 x_3,
\end{aligned}
\tag{1}
$$

where $\alpha_y = 0.8$, $\beta_1 = 0.6$, $\beta_2 = 0.8$, $\alpha_z = 0.8$, $\gamma_1 = 0.4$, $\gamma_2 = 0.6$.

For strategy (1), we define the conditional Granger causality from time-course X to Z conditional on time-course Y as,

$$F_{X\to Z|Y} = \ln \frac{\|e(Z_{\{Y\}})\|}{\|e(Z_{\{X,Y\}})\|},$$

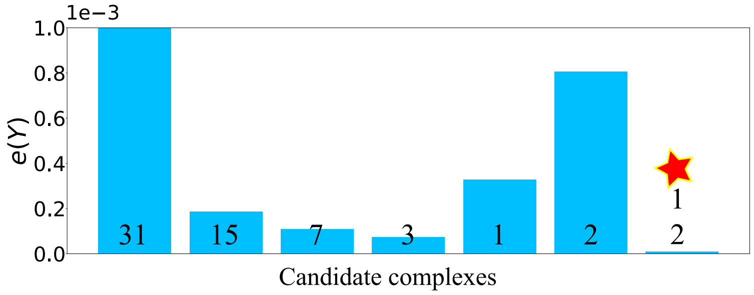

Supplementary Fig. 3: Higher-order neighbors inference results of the node Y.

where $\|e(Z_{\mathcal{C}})\|$ represents the average prediction error of node Z with the candidate neighbor set $\mathcal{C}$ (see Section 4.2 for details). According to this definition, when $F_{\mathcal{C}_1\to Z|\mathcal{C}_2} \approx 0$, $\{X_1, X_2\}$ has no direct effect on Z, whereas when $F_{\mathcal{C}_1\to Z|\mathcal{C}_2} > 0$, $\{X_1, X_2\}$ has a direct effect on Z. By conducting the experiment on the system of Eq. (1), we have $F_{\mathcal{C}_1\to Z|\mathcal{C}_2} = -0.043 \approx 0$ and $F_{\mathcal{C}_2\to Z|\mathcal{C}_1} = 2.983$. Therefore, X_1 and X_2 are indirect causes of Z, while Y is a direct cause of Z.

For strategy (2), we can further consider the direct causal links between $\{X_1, X_2\}$ and $\{Y\}$ to eliminate the indirect causal cases, $\{X_1, X_2\} \to \{Y\} \to \{Z\}$ or $\{Y\} \to \{X_1, X_2\} \to \{Z\}$. This is because when both $\{X_1, X_2\}$ and $\{Y\}$ can independently predict Z, then only one of them is a direct cause, while the other is an indirect cause. More precisely, if one can accurately infer that $O_Y = \{X_1, X_2\}$, then Y is a direct neighbor of Z. In contray, if $O_{X_1} = O_{X_2} = Y$, then X_1 and X_2 are direct neighbors of Z. By conducting an experiment on the system described by Eq. (1), the results of inferring higher-order neighbors of node Y are shown in Supplementary Fig. 3. It is observed that the prediction error of node Y using the $\{X_1, X_2\}$ is remarkably small. This strongly suggests that $\{X_1, X_2\}$ is a direct cause of Y, thereby implying that Y is the direct neighbor of Z.

In summary, our framework can effectively infer the structure of dynamical systems and can be extended to identify the direct and indirect causality.

Supplementary Note 2. Effects in Other Common Differential Dynamical Systems

2.1. Effects in several common chaotic systems

To verify the effect of the HoGRC method in other systems, we conduct experiments in several chaotic systems in this section. The hyperparameters of the reservoirs in all experiments are given in Section 3. Similar to the metric VPS in the main text, we define the valid prediction time (VPT) as $\text{VPT} = \inf\{\lambda_1 t : \text{RMSE}(t) > \epsilon_r\}$ for the classical chaotic dynamical systems, where λ_1 is the largest Lyapunov exponent (LE), and ϵ_r is the positive threshold.

In addition, to facilitate processing, we designate all possible higher-order terms as candidate simplices, denoting them in the binary form $b_{x_N} \cdots b_{x_2} b_{x_1}$ (where $b_{x_i} = 1$ if the complex contains node x_i and 0 otherwise), which can be labeled using decimal numbers

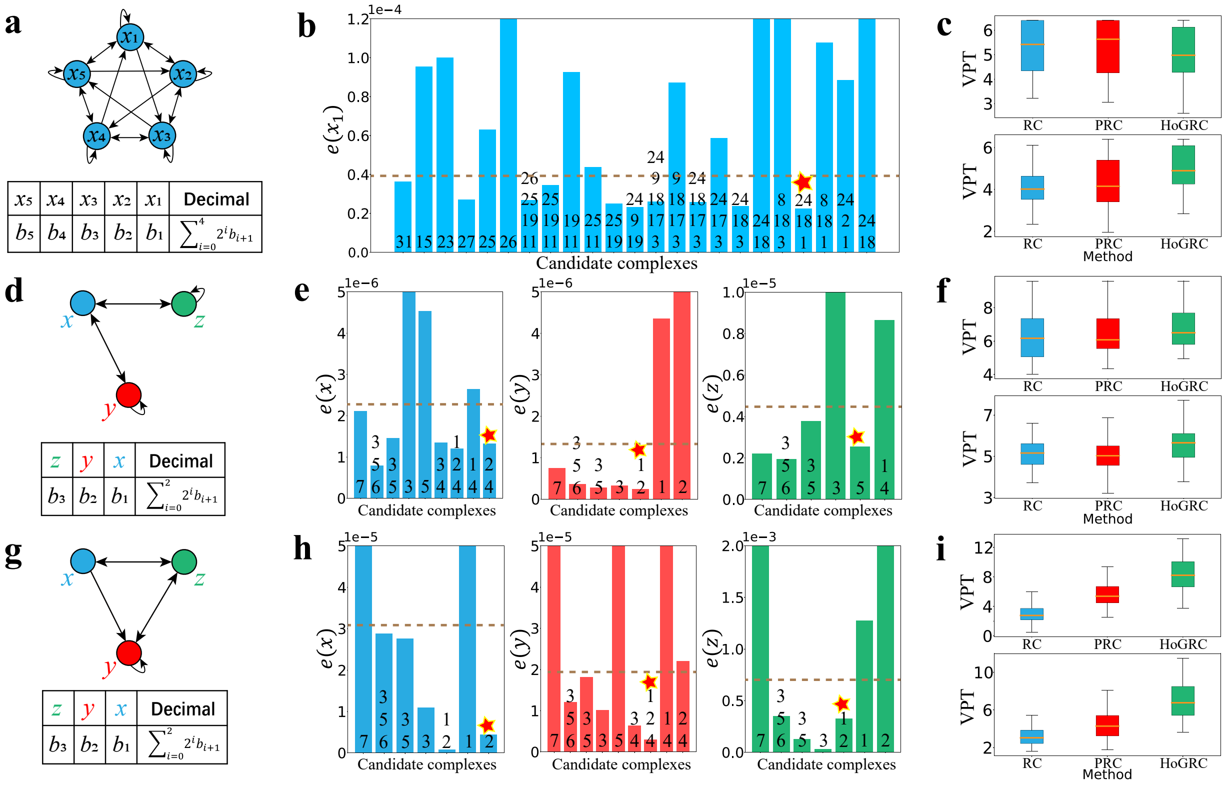

Supplementary Fig. 4: Higher-order structure inference and data prediction for the Lorenz96, the Rossler and the Colpitts systems. a-c Inference of higher-order neighbors and data prediction for the Lorenz96 system. **d-f** Inference of higher-order neighbors and data prediction for the Rossler system. **g-i** Inference of higher-order neighbors and data prediction for the Colpitts system. Also, for the Lorenz96 system, we generate data with $T = 5000$ and $\Delta t = 0.02$. For the Rossler and Colpitt system, we take $T = 5000$ and $\Delta t = 0.2$. In subfigures c, f and i, the orange line in the middle of the box represents the median, the upper and lower boundaries of the box represent the upper and lower quartiles, respectively. The boundaries of the upper and lower whiskers represent the maxima and minima, respectively.

$(2^{N-1}b_{x_N} + \cdots + 2^1 b_{x_2} + 2^0 b_{x_1})$. For example, the candidate complexes for the Lorenz63 system correspond to the decimal numbers from 1 to 7, namely $\{x\}$, $\{y\}$, $\{x, y\}$, $\{z\}$, $\{x, z\}$, $\{y, z\}$, and $\{x, y, z\}$.

As shown in Supplementary Fig. 4a, we first consider the Lorenz system proposed in 1996 (Lorenz96) [2], and its dynamic equations are

$$\dot{x}_i = x_{i-1}(x_{i+1} - x_{i-2}) - x_i + f, \tag{2}$$

where $i = 1, 2, \cdots, N$, $x_{-1} = x_{N-1}$, $x_0 = x_N$, $x_{N+1} = x_1$. We take $f = 8.0$ and $N = 5$ to generate experimental data and divide the training set and testing set in the same way. At this point, we number the possible candidate complexes with the decimal number $\sum_{i=0}^{4} 2^i b_{i+1}$. For node x_1, its higher-order neighbors are $\{\{x_1\}, \{x_4, x_2\}, \{x_4, x_3\}\}$, and the corresponding decimal number are $\{24, 18, 1\}$. Supplementary Fig. 4b shows the inference

process of node x_1, and choosing an appropriate threshold can infer the true higher-order neighbors. And Supplementary Fig. 4c shows that our method has the best prediction performance. Similarly, as shown in Supplementary Fig. 4d, we consider the Rossler system [3], and the equations are

$$\begin{aligned} \dot{x} &= -(y+z), \\ \dot{y} &= x + \alpha y, \\ \dot{z} &= \beta + z(x-\gamma). \end{aligned} \tag{3}$$

We take $\alpha = 0.2$, $\beta = 0.2$, $\gamma = 5.7$ and $N = 3$ to generate experimental data. Supplementary Figs. 4e-f show the inference results of higher-order neighbors and multi-step prediction effects, respectively. In addition, we consider the Colpitts system [4] describing chaos in a nonlinear circuit (see Supplementary Fig. 4g). The equations are

$$\begin{aligned} \dot{x} &= \alpha y, \\ \dot{y} &= -\gamma(x+z) - qy, \\ \dot{z} &= \eta(y + 1 - \exp(-x)). \end{aligned} \tag{4}$$

We take $\alpha = 5$, $\gamma = 0.0797$, $q = 0.6898$, $\eta = 6.2723$ and $N = 3$ to generate experimental data. Supplementary Figs. 4h-i show the inference results of higher-order neighbors and multi-step prediction effects, respectively.

The experimental results of the above three chaotic systems show that our HoGRC method can infer higher-order dynamical relationships and achieve long-term predictions.

2.2. The hyperchaotic system with four wings

When a nonlinear oscillator has two large positive Lyapunov exponents, it is often regarded as a hyperchaotic system. Here, to further illustrate the efficacy of our method, we use the data produced by such a hyperchaotic system as:

$$\begin{aligned} \dot{x} &= a(y-x) - dz - ku, \\ \dot{y} &= b(x+y) - dz - xzw, \\ \dot{z} &= ey - cz + xyw, \\ \dot{w} &= xyz - dw, \\ \dot{u} &= ryz + pu. \end{aligned} \tag{5}$$

This system, with the parameters set as $a = 35$, $b = 13$, $c = 10$, $d = 12$, $e = 10$, $k = 0.01$, $r = 0.001$, and $p = -0.001$, can produce four wings constituting a hyper-chaotic attractor (see the trajectories wandering in the phase spaces in Supplementary Fig. 5a) [5], where the largest two LE's are $\lambda_1 = 14.603$ and $\lambda_2 = 7.948$. Supplementary Fig. 5b shows the inference procedure for the higher-order neighbors of the node x, where the binary labels of x, y, z, w, u, respectively, are $b_1, b_2, \cdots, b_5$, so the decimal representation of the candidate complex is $\sum_{i=0}^{4} 2^i b_{i+1}$. Clearly, our method accurately infers the higher-order neighbors of the node z, and the remaining inference procedures are shown in Supplementary Fig. 6. As clearly seen in Supplementary Figs. 5c-d, our HoGRC method does outperform all the other methods.

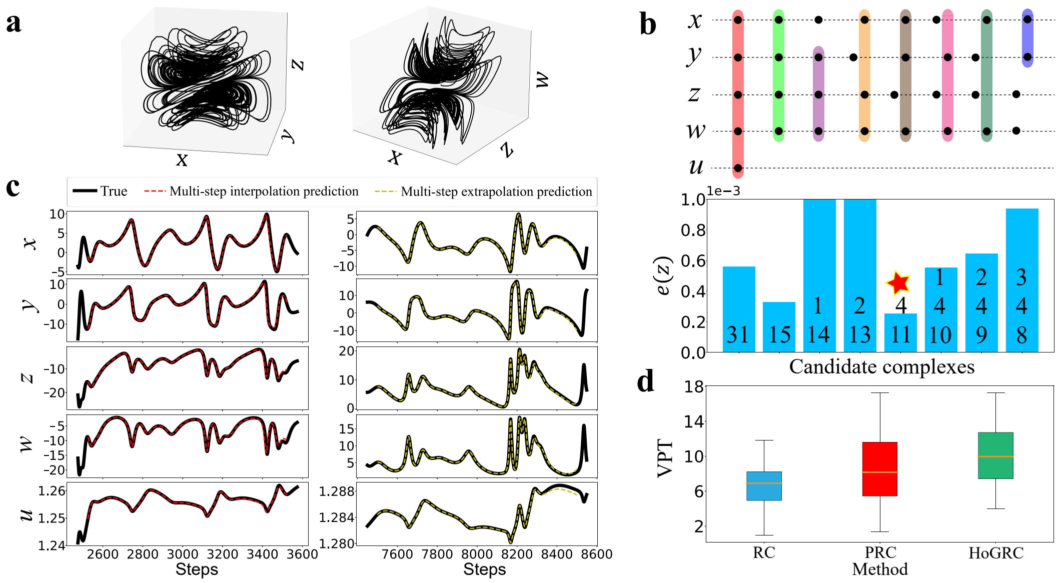

Supplementary Fig. 5: Higher-order neighbors inference and dynamics prediction for the hyperchaotic system with four wings. **a** Dynamics of system (5) in different phase spaces, the x-y-z and the x-z-w projections. **b** higher-order neighbors inference of the node z. **c** Prediction results on different system variables using the HoGRC method. **d** Prediction results applying different methods to the testing data. The orange line in the middle of the box represents the median, the upper and lower boundaries of the box represent the upper and lower quartiles, respectively. The boundaries of the upper and lower whiskers represent the maxima and minima, respectively. Here, we generate the experimental data with $\Delta t = 0.001$ and $T = 10000$.

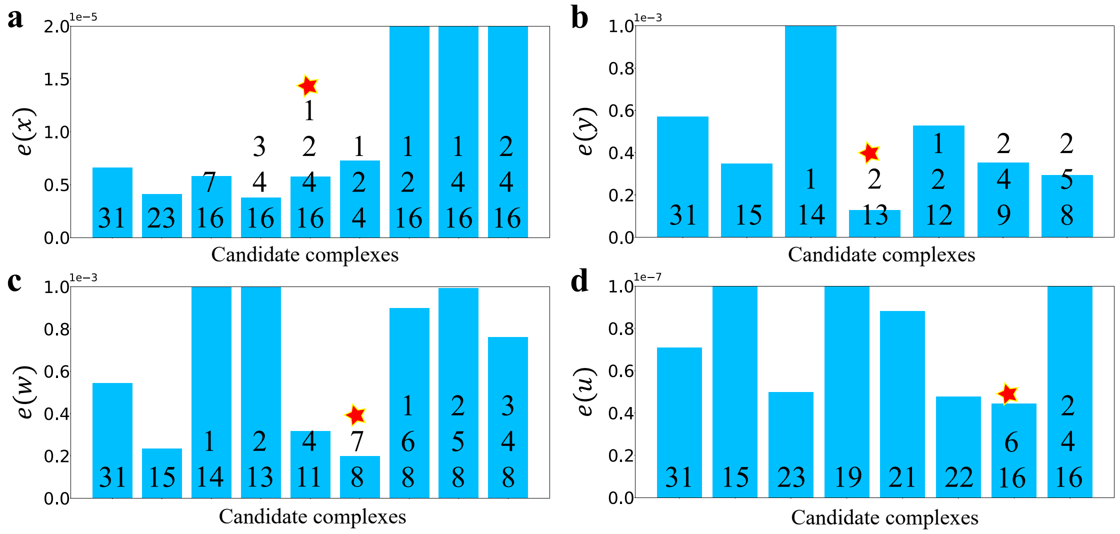

Supplementary Fig. 6: Higher-order neighbor inference for the NFW systems. **a-d** Figures a,b,c and d are the higher-order neighbor inference process of nodes x, y, w, u, respectively.

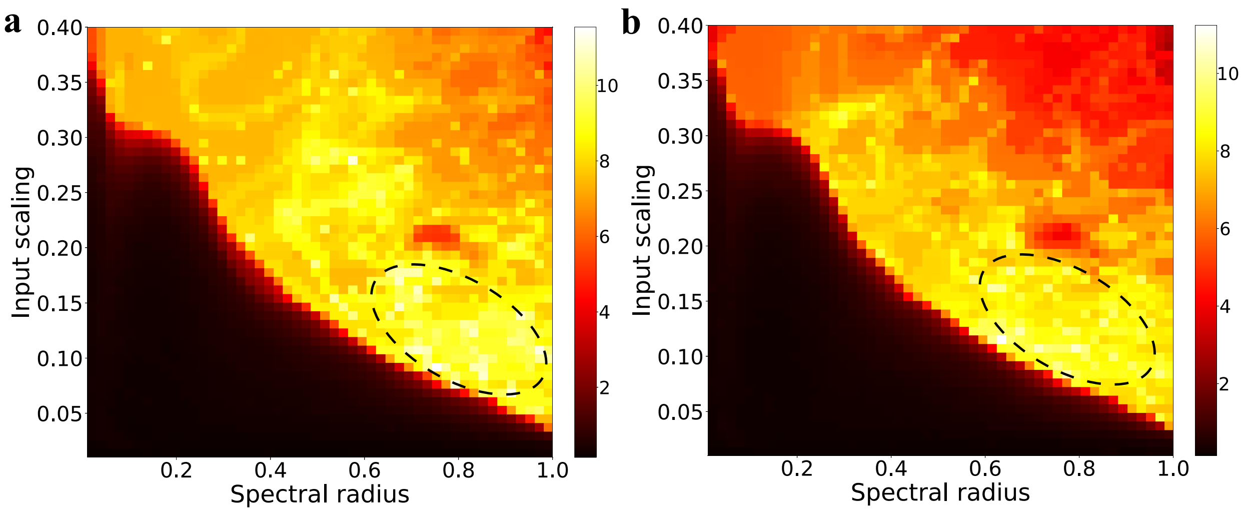

Supplementary Fig. 7: Heatmaps of valid prediction time at different spectral radius and input scaling. **a** Interpolated predictions on the training set. **b** Extrapolated predictions on the testing set.

Supplementary Table 1: Hyperparameters for the experiments of the Lorenz63 system.

	n	t_{w}	c_{o}	l	r	s	σ_{b}	λ_W
RC	1000	100	0.02	0.01	0.85	0.1	1	10^{-9}
PRC	1000	100	0.02	0.05	0.85	0.1	1	10^{-8}
HoGRC	1000	100	0.02	0.05	0.85	0.1	1	10^{-8}

Supplementary Note 3. Hyperparameter settings for different models

The hyperparameters in the reservoir have a significant impact on the final prediction performance. Therefore, for different systems, it is necessary to select appropriate parameters accordingly, including the number of nodes in the reservoir network n, the connectivity c_{o}, the number of warm-up steps t_{w}, the spectral radius r, the input scaling s, the leaky rate l, the bias term σ_{b}, the ridge regression regularization coefficient λ_W.

For the experiments of the Lorenz63 system (Fig. 2 in the main text), the optimal RC hyperparameters we set are shown in Supplementary Table 1. Parameter $N_{\mathrm{r}} = n \times N$ determines the dimension of the hidden state. Generally speaking, a larger n can improve the learning ability of the method. Of course, the promotion effect of larger n will also tend to be saturated. Therefore, its value is also related to the size of the training set. Parameter c_{o} determines the connectivity of the reservoir network, usually a larger connectivity will enhance the nonlinear approximation ability of the method. However, for most experiments, a sparse structure will usually give better results. Parameter s is a factor to scale the input signal, and parameter r weights the largest eigenvalue of the adjacency matrix. These two parameters are the key optimization parameters of RC, as shown in Supplementary Fig. 7, we conduct multiple experiments to search for the optimal parameter values, and there is an optimal region (outlined in a dashed circle). The remaining parameters can also be searched

Supplementary Table 2: Hyperparameters for the experiments of the CL63 system.

	n	t_{w}	c_{o}	l	r	s	σ_{b}	λ_W
RC (linear)	1000	100	0.02	0.1	0.8	0.01	1	10^{-8}
PRC (linear)	1000	100	0.02	0.1	0.8	0.02	1	10^{-8}
HoGRC (linear)	1000	100	0.02	0.1	0.8	0.02	1	10^{-8}
RC (trigonometric)	1000	100	0.02	0.4	0.8	0.05	2	10^{-2}
PRC (trigonometric)	1000	100	0.02	0.4	0.8	0.02	1	10^{-3}
HoGRC (trigonometric)	1000	100	0.02	0.4	0.8	0.02	1	10^{-3}
RC (absolute)	1000	100	0.05	0.5	0.9	0.1	1	10^{-3}
PRC (absolute)	1000	100	0.05	0.1	0.9	0.2	0.8	10^{-2}
HoGRC (absolute)	1000	100	0.05	0.1	0.9	0.2	0.8	10^{-2}

Supplementary Table 3: Hyperparameters for the experiments of the Lorenz96, Rossler and Colpitts systems.

	n	t_{w}	c_{o}	l	r	s	σ_{b}	λ_W
RC (Lorenz96)	1000	100	0.02	0.1	0.9	0.06	0.8	10^{-9}
PRC (Lorenz96)	1000	100	0.02	0.1	0.9	0.06	0.8	10^{-9}
HoGRC (Lorenz96)	1000	100	0.02	0.1	0.9	0.06	0.8	10^{-9}
RC (Rossler)	1000	100	0.02	0.01	0.5	0.2	2.5	10^{-8}
PRC (Rossler)	1000	100	0.02	0.01	0.8	0.3	1	10^{-9}
HoGRC (Rossler)	1000	100	0.02	0.01	0.8	0.3	1	10^{-9}
RC (Colpitts)	1000	100	0.02	0.3	0.6	0.2	1	10^{-6}
PRC (Colpitts)	1000	100	0.02	0.1	0.6	0.5	1	10^{-8}
HoGRC (Colpitts)	1000	100	0.02	0.1	0.6	0.5	1	10^{-8}

to select reasonable values.

For the experiments of the CL63 system (Fig. 2 in the main text), the optimal RC hyperparameters we set are shown in Supplementary Table 2. Linear, trigonometric, and absolute denote experiments where the coupling terms are $(y_j - y_i)$, $sin(y_j - y_i)$ and $|y_j - y_i|$, respectively. For the experiments of the chaotic systems (see Supplementary Fig. 4), the optimal RC hyperparameters we set are shown in Supplementary Table 3. For the experiments of the NFW system (see Supplementary Fig. 5), the optimal RC hyperparameters we set are shown in Supplementary Table 4. For the experiments of the network systems (Fig. 3 in the main text), the optimal RC hyperparameters we set are shown in Supplementary Table 5. For the experiments of the power grid system (Fig. 4 in the main text), the optimal RC hyperparameters we set are shown in Supplementary Table 6. Finally, for the experiment in Fig. 5 in the main text, the experiments after adding noise only need to increase the regularization coefficient λ_W appropriately, and the other parameters are the same as Supplementary Tables 1, 2 and 4.

Supplementary Table 4: Hyperparameters for the experiments of the NFW system.

	n	t_{w}	c_{o}	l	r	s	σ_{b}	λ_W
RC	1000	100	0.02	0.05	0.85	0.1	1	10^{-6}
PRC	1000	100	0.02	0.03	0.85	0.1	1	10^{-8}
HoGRC	1000	100	0.02	0.03	0.85	0.1	1	10^{-8}

Supplementary Table 5: Hyperparameters for the experiments of the network dynamical systems.

	n	t_{w}	c_{o}	l	r	s	σ_{b}	λ_W
RC (FHNS)	1000	100	0.05	0.1	0.65	1	1.2	10^{-8}
PRC (FHNS)	500	100	0.05	0.1	0.55	1	1.2	10^{-8}
HoGRC (FHNS)	500	100	0.05	0.1	0.55	2	1.2	10^{-8}
RC (CRoS)	1000	100	0.02	0.05	0.8	0.1	3	10^{-8}
PRC (CRoS)	500	100	0.02	0.1	0.8	0.05	2.5	10^{-8}
HoGRC (CRoS)	500	100	0.02	0.1	0.8	0.1	2.5	10^{-8}
RC (CsH^{2}S)	1000	100	0.05	0.3	0.8	0.4	1	10^{-3}
PRC (CsH^{2}S)	500	100	0.05	0.08	0.8	0.4	1	10^{-4}
HoGRC (CsH^{2}S)	500	100	0.05	0.08	0.8	0.4	1	10^{-5}

Supplementary Table 6: Hyperparameters for the experiments of the power grid system.

	n	t_{w}	c_{o}	l	r	s	σ_{b}	λ_W
RC	2000	100	0.02	0.1	0.8	0.5	1	10^{-4}
PRC	2000	100	0.02	0.1	0.8	0.5	1	10^{-4}
HoGRC	2000	100	0.02	0.1	0.8	0.5	1	10^{-4}

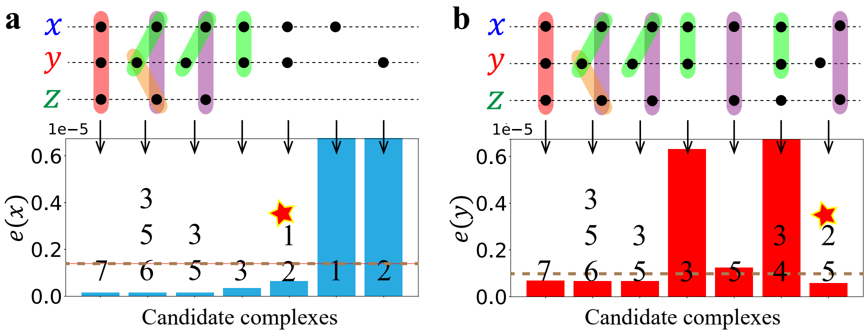

Supplementary Fig. 8: Higher-order neighbors inference results in Lorenz system. **a** Higher-order neighbors inference of node x. **b** Higher-order neighbors inference of node y. Here, the red pentagrams indicates inferred higher-order neighbors.

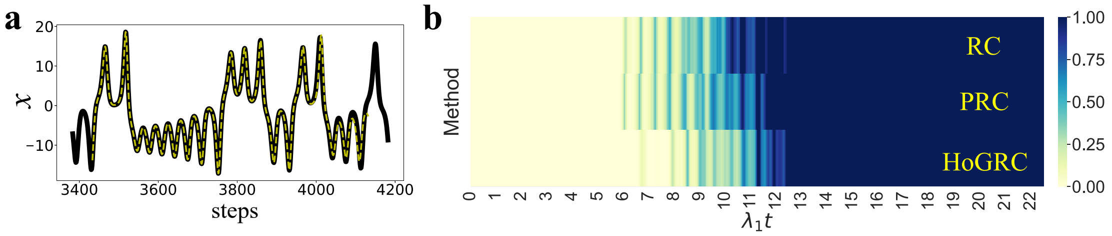

Supplementary Fig. 9: Performance of HoGRC method in Lorenz63 prediction task. **a** the extrapolation prediction effect of our method on the x dimension, where the black line represents the true value and the yellow line represents the predicted value. **b** The average error of different methods.

Supplementary Note 4. Some Supplementary Experiments

In this section, we conduct supplementary experiments to complement those presented in the main text.

4.1. Lorenz63 system

We present the structural inference process for the nodes x and y of the Lorenz63 system in Supplementary Figs. 8a and 8b, analogous to Fig. 2a in the main text. It is evident that our proposed method can accurately infer the higher-order structure of the Lorenz63 system.

In addition, due to the dynamic form of the single Lorenz63 system being relatively simple, different methods can achieve great predictions. Supplementary Fig. 9a shows the extrapolation prediction effect of our method on the x dimension, and Supplementary Fig. 9b compares the prediction errors of various methods.

4.2. Lyapunov exponents of the coupled Lorenz63 system

We use the Matlab toolbox to calculate the Lyapunov exponent of the coupled Lorenz63 system, and the calculation results are shown in Supplementary Fig. 10. Six of the Lyapunov exponents are greater than zero, with values of 12.42, 9.97, 8.12, 3.29, 1.35, and 0.02.

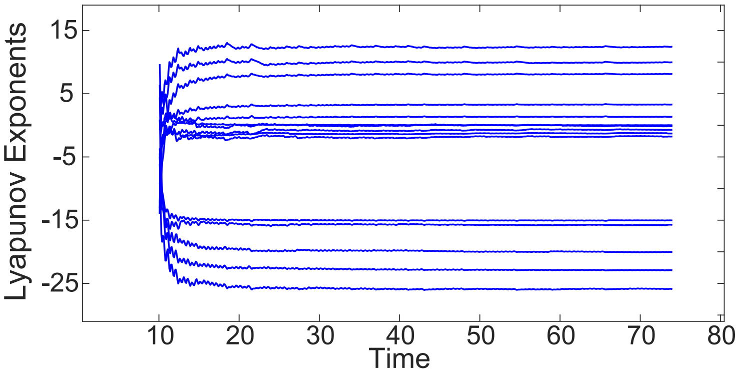

Supplementary Fig. 10: Lyapunov exponents of the coupled Lorenz63 system.

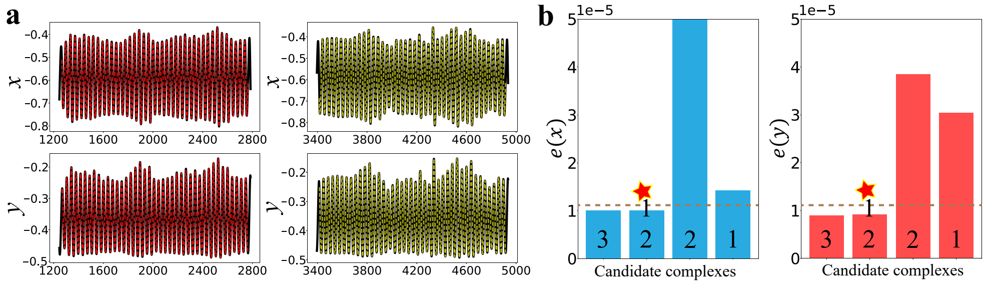

Supplementary Fig. 11: Predictive effect and higher-order neighbor inference of the FHNS systems. **a** Prediction performance of the HoGRC method in the FHNS system. **b** Higher-order neighbor inference for the FHNS system.

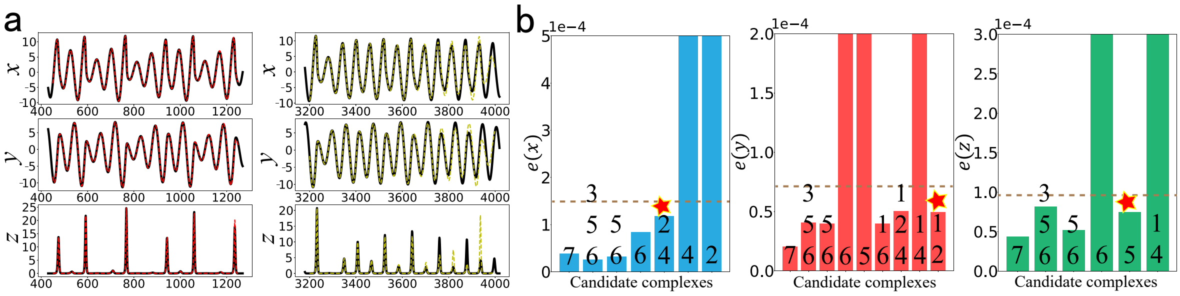

Supplementary Fig. 12: Predictive effect and higher-order neighbor inference of the CRoS systems. **a** Prediction performance of the HoGRC method in the CRoS system. **b** Higher-order neighbor inference for the CRoS system.

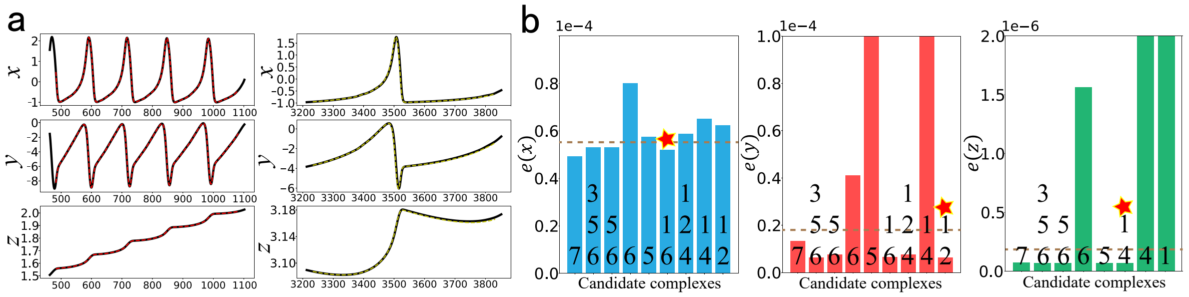

Supplementary Fig. 13: Predictive effect and higher-order neighbor inference of the CsH^{2}S systems. **a** Prediction performance of the HoGRC method in the CsH^{2}S system. **b** Higher-order neighbor inference for the CsH^{2}S system.

4.3. Higher-Order Neighbor Inference in Network Dynamical Systems

In addition, in the experiment of the network dynamical system (Fig. 3 of the main text), we only give the inference process of the coupled network, and we introduce the inference process of the higher-order neighbors below. Supplementary Fig. 11a shows the prediction performance of the HoGRC method in the FHNS systems, and Supplementary Fig. 11b shows the higher-order neighbor inference for FHNS systems. Similarly, we show the experimental results of the CRoS system and CsH^{2}S system in Supplementary Fig. 12 and Supplementary Fig. 13, respectively. We found that choosing an appropriate threshold ϵ_e can accurately infer the higher-order neighbors of all nodes. However, in the CsH^{2}S system, we misjudged the higher-order neighbors $\{1, 2, 4\}$ of node x as $\{1, 6\}$. There are two main reasons for our analysis. First, the candidate complex set $\{1, 2, 3\}$ is very close to $\{1, 6\}$, and $\{1, 2, 3\} \subset \{1, 6\}$, so using $\{1, 6\}$ as higher-order neighbors can also make better multi-step predictions. Second, the degree of nonlinearity of the CsH^{2}S system is higher, especially the coupling term. That is, the stronger the predictive power of HoGRC and the more accurate the modeled dynamical system, the easier it is to infer higher-order neighbors.

4.4. Structure information of the UK power grid

In the main text, we verify our method with the high-order Kuramoto model on a real power grid structure. The global structure of the network is shown in Fig. 4 of the main text. In addition, we also add some higher-order interactions (using $\{i, j, k\}$ to represent $B_{ijk} = 1$) from the distinct triangles in the UK power grid, which are $\{14, 111, 112\}$, $\{33, 30, 32\}$, $\{49, 57, 114\}$, $\{81, 82, 117\}$, $\{84, 87, 88\}$, and $\{100, 95, 109\}$ respectively.

4.5. System structure inference with noise data

We set the noise intensity $\sigma_n = 0.2$, and Supplementary Fig. 14 shows the structural inference of the Lorenz63 and CL63 systems in the case of noisy data. Supplementary Fig. 14a shows the higher-order neighbor inference process of the Lorenz63 system, and Supplementary Fig. 14b shows the inference process of the coupled network of the CL63 system.

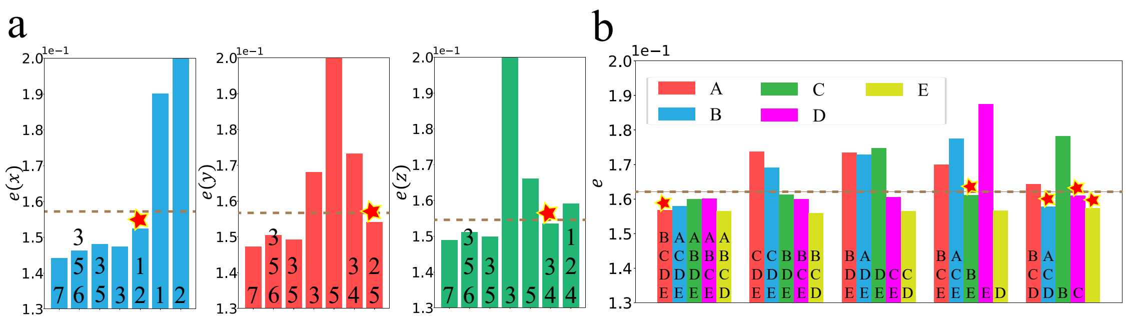

Supplementary Fig. 14: Structural inference with noisy data. a Infer the higher-order neighbor of the Lorenz63 system with noise intensity $\sigma_{\rm n} = 0.2$. **b** Infer the coupling network of the CL63 system with noise intensity $\sigma_{\rm n} = 0.2$.

4.6. Influence of coupling strength

Indeed, the coupling strength γ is also an important parameter in experiments. As mentioned in Section 2.4 of the main text: A very small coupling strength implies a weak coupling effect on the dynamics, while sufficiently strong coupling tends to increase predictability due to a high probability of synchronization occurrence. Therefore, in our experiments, we purposely selected a moderate level of coupling strength to increase the prediction difficulty.

To verify the aforementioned statement, we conducted additional experiments by varying the coupling strengths in the coupled Rössler system (CRoS). As depicted in Supplementary Fig. 15a, it is observed that the lower coupling strength does not significantly affect the predictive performance of our method. However, upon exceeding a certain threshold, the subsystems exhibit synchronization. The dynamics thereby becomes extremely predictable. Furthermore, Supplementary Figs. 15b-d demonstrate that the higher coupling strengths are associated with easier coupled structure inference tasks. Specifically, Supplementary Fig. 15d provides clear evidence that the coupling neighbors of node B are $\{A, C, D\}$ (refer to Definition 3 in the manuscript for the detailed inference strategy). Conversely, when the coupling strength is low, as depicted in Supplementary Fig. 15b ($\gamma = 0.01$), the difference in metrics $e(B)$ for various candidate structures decreases, thereby increasing the difficulty of the inference task. Nevertheless, as HoGRC is a delicate method by integrating the structures, one can still accurately identify the coupling structure by choosing a smaller value of ϵ_e in Definition 3 of the main text. However, it is worth noting that this choice may inevitably reduce the robustness of the inference.

4.7. Higher-order Kuramoto model with phase lags

In power systems, transmission losses are an inherent and unavoidable phenomenon that occurs due to the resistance in the conducting materials. One approach to approximate these losses is the use of a Kuramoto model with phase lag α, as described in reference [6]. In this section, we similarly introduce the phase lags α and β to obtain a higher-order

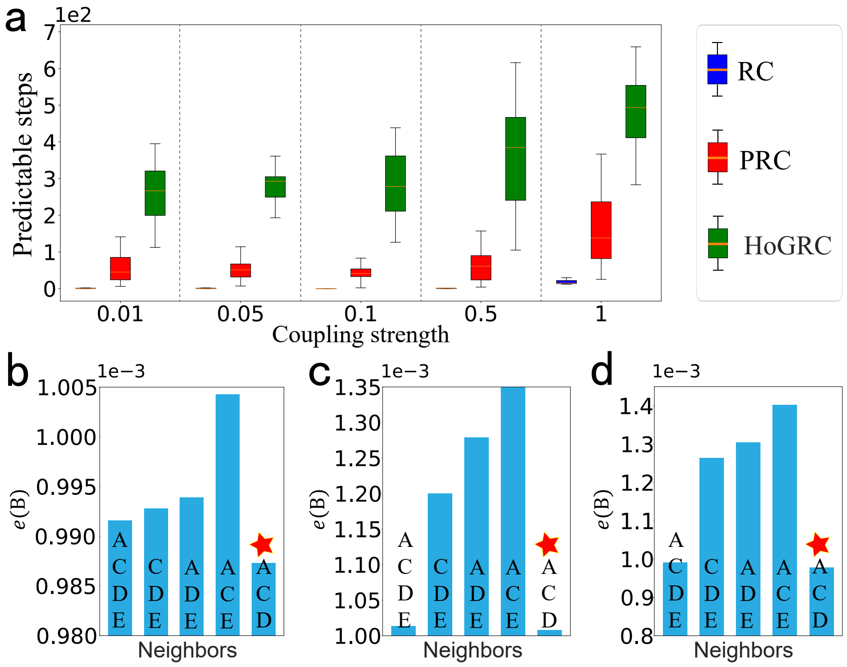

Supplementary Fig. 15: Experimental results of the HoGRC method under different coupling strengths in CRoS. a The Performance of extrapolation prediction under different coupling strengths. The orange line in the middle of the box represents the median, the upper and lower boundaries of the box represent the upper and lower quartiles, respectively. The boundaries of the upper and lower whiskers represent the maxima and minima, respectively. **b-d** The structural inference of node B under different coupling strengths. Here, the value of γ is set to 0.01, 0.1, and 1, respectively.

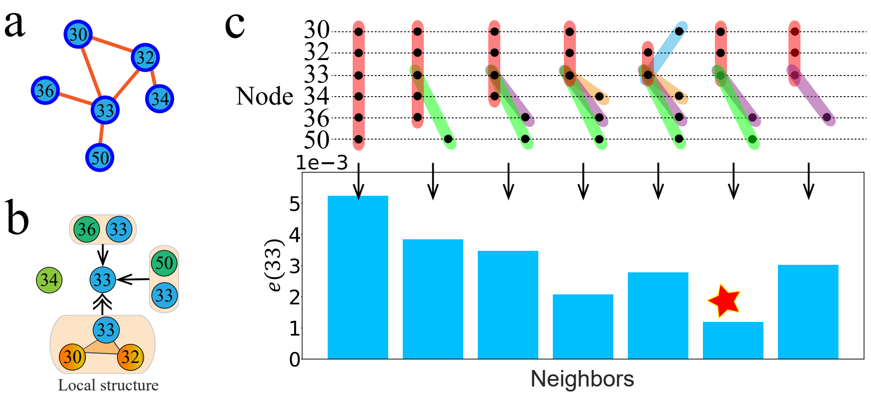

Supplementary Fig. 16: Higher-order neighbors inference in the higher-order Kuramoto model with phase lags. a The local location of 33 in UK power grid. **b** Local coupling structure of node 33 in networks. **c** Higher-order neighbors inference of node 33.

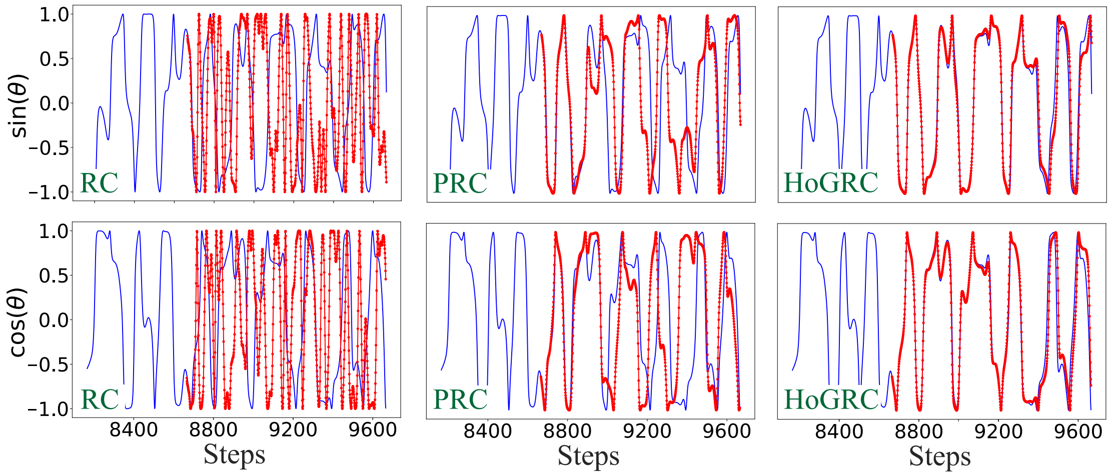

Supplementary Fig. 17: Dynamics prediction in the higher-order Kuramoto model with phase lags. These figures show the extrapolation prediction of node 33 under different methods, with the true value shown in blue and the predicted value in red.

Kuramoto model with phase lags, given by

$$\dot{\theta}_i = \omega_i + \gamma_1 \sum_{j=1}^{N} A_{ij} \left[\sin(\theta_j - \theta_i - \alpha) + \sin(\alpha)\right] + \gamma_2 \sum_{j=1}^{N} \sum_{k=1}^{N} B_{ijk} \left[\sin(\theta_j + \theta_k - 2\theta_i - \beta) + \sin(\beta)\right].$$

In our experiment, we set α and β to be 0.05 and 0.10, respectively. Similarly, we conduct experiments on the tasks of structure inference and dynamics prediction in the main text, and the experimental parameters are kept consistent with those described in the main text. As shown in Supplementary Fig. 16 and Supplementary Fig. 17, it is evident that our method maintains optimal predictive performance in experiments of power grids with phase lags, while also inferring higher-order structures of nodes based on observed data.

Supplementary Note 5. NDCN and TPI Methods for Network Dynamical Systems

The literature [7] proposed the NDCN method, which considers a combination of graph neural networks (GNN) and neural differential equations to predict network dynamics. First, the time series data $X(t)$ is encoded into the hidden layer state $X_{\mathrm{h}}(t) = f_{\mathrm{e}}(X(t), W_{\mathrm{e}})$ through the neural network, and then we model the hidden layer dynamics through a graph neural network:

$$X_{\mathrm{h}}(t + \Delta t) = X_{\mathrm{h}}(t) + \int_t^{t+\Delta t} f(X_{\mathrm{h}}, G, W_G, \tau)$$

where G is the network structure and f is the GNN mapping. Finally, we decode the hidden state into the original space and make a prediction $\hat{X}(t) = f_d(X_{\mathrm{h}}(t), W_{\mathrm{d}})$. The parameters

that need to be trained during the training process are W_e, W_G and W_d. We follow the loss function in Ref. [7], and take the epoch equal to 200 and the learning rate equal to 0.01 for training. However, the experiments in this paper cannot achieve good results. The main reason for our analysis is that the systems involved in this paper have more complex chaotic characteristics, but this method is suitable for complex network systems that tend to equilibrium.

For the prediction problem of network dynamics, Reference [8] presents a new solution (we call it TPI). They first define a library of fundamental functions, including polynomial, trigonometric, exponential, fractional, rescaling, and various activation functions. It is then trained on known time series data and uses linear combinations of these basis functions to model system dynamics. However, due to the small amount of experimental data of the network dynamic system involved in this paper, and the self-dynamics of different nodes and the interactive dynamics between nodes are not the same, this method also performs poorly.

In addition, using the above two methods needs to know the spatial structure information of the system in advance. In contrast, our method can not only infer the underlying coupling network, but also make longer-term predictions. Therefore, the HoGRC method also has advantages in the prediction problem of network dynamics.

References

[1] Xinyu Han, Yi Zhao, and Michael Small. A tighter generalization bound for reservoir computing. *Chaos: An Interdisciplinary Journal of Nonlinear Science*, 32(4):043115, 2022.

[2] Edward N Lorenz. Predictability: A problem partly solved. In *Proc. Seminar on Predictability*, volume 1, 1996.

[3] Otto E Rössler. An equation for continuous chaos. *Physics Letters A*, 57(5):397–398, 1976.

[4] Jason A Platt, Stephen G Penny, Timothy A Smith, Tse-Chun Chen, and Henry DI Abarbanel. A systematic exploration of reservoir computing for forecasting complex spatiotemporal dynamics. *arXiv preprint arXiv:2201.08910*, 2022.

[5] Wenbo Liu and Guanrong Chen. A new chaotic system and its generation. *International Journal of Bifurcation and Chaos*, 13(01):261–267, 2003.

[6] Frank Hellmann, Paul Schultz, Patrycja Jaros, Roman Levchenko, Tomasz Kapitaniak, Jürgen Kurths, and Yuri Maistrenko. Network-induced multistability through lossy coupling and exotic solitary states. *Nature communications*, 11(1):592, 2020.

[7] Chengxi Zang and Fei Wang. Neural dynamics on complex networks. In *Proceedings of the 26th ACM SIGKDD International Conference on Knowledge Discovery and Data Mining*, page 892–902, 2020.

[8] Ting-Ting Gao and Gang Yan. Autonomous inference of complex network dynamics from incomplete and noisy data. *Nature Computational Science*, 2(3):160–168, 2022.
